# Detection systems for antibody responses against herpes B virus

**DOI:** 10.5194/pb-4-9-2017

**Published:** 2017-02-17

**Authors:** Stefan Pöhlmann, Astrid Krüger, Wali Hafezi, Stefan Schneider, Jens Gruber, Michael Winkler, Artur Kaul

**Affiliations:** 1 Infection Biology Unit, German Primate Center, 37077 Göttingen, Germany; 2 Department of Medical Microbiology, University of Münster, 48149 Münster, Germany; 3 Primate Genetics Laboratory, German Primate Center, 37077 Göttingen, Germany

## Abstract

Herpes B virus (BV) infection is highly prevalent among adult Asian macaques and rarely causes severe disease in infected animals. In contrast, BV infection of humans can induce fatal encephalitis in the absence of treatment. Therefore, the development of diagnostic tests for specific and sensitive detection of antibodies against BV is an important task. The cross-reactivity of antibodies against BV with related simplex viruses of other primates may afford an opportunity to obtain sensitive detection systems without the need to work with the highly pathogenic BV. Moreover, it has been proposed that use of recombinant viral glycoproteins may allow for a detection of antibody responses against BV with high specificity. However, limited data are available for both approaches to BV diagnostic. Here, we report that simian agent 8 (SA8; infects African green monkeys)- and herpesvirus papio 2 (HVP-2; infects baboons)-infected cells allow for a more sensitive detection of antibody responses against BV in macaques than lysates of herpes simplex virus type 1 and 2 (HSV-1/2; infect humans)-infected cells and a commercial HSV ELISA (Enzygnost^®^ Anti-HSV/IgG). In addition, we show that sera from BV-infected macaques frequently contain antibodies against the recombinant BV glycoprotein gD (BV gD) that has been previously proposed as a diagnostic target for discriminating BV- and HSV-induced antibodies. However, we found that antibodies of some HSV-infected human patients also reacted with BV gD. In contrast, only sera of HSV-1- and HSV-2-infected humans, but not sera from BV-infected macaques, reacted with HSV-1/2 gG. Collectively, these results suggest that both SA8 and HVP-2 allow for sensitive and comparable detection of BV-directed antibody responses in macaques and that the combination of BV gD and HSV-1/2 gG needs to be complemented by a least one additional viral glycoprotein for reliable discrimination between antibody responses against BV and HSV-1/2 in humans.

## 1 Introduction

Herpesviruses are large, enveloped DNA viruses that infect diverse vertebrate and invertebrate hosts. A hallmark of herpesviruses is the latent infection of certain host cells, in which the viruses can persist in a dormant form for long time periods (Koyuncu et al., 2013). Members of the genus *Simplexvirus* within the subfamily Alphaherpesvirinae infect humans and non-human primates (NHPs) but infection is usually not associated with severe disease. For instance, herpes simplex virus type 1 (HSV-1) and HSV-2 infect cells in the oral and genital mucosa and may cause lesions in these tissues, which usually heal without scarring (Delaney et al., 2014; Xu et al., 2006). However, HSV-1 and HSV-2 infection rarely causes encephalitis or other serious complications despite the pronounced neurotropism and high prevalence of both viruses in the human population (Delaney et al., 2014; Xu et al., 2006). Similarly, herpes B virus (BV, *Macacine herpesvirus 1*, also termed B virus, herpesvirus simiae or *Cercopithecine herpesvirus 1*), the HSV homologue of macaques (Elmore and Eberle, 2008; Hilliard, 2007; Huff and Barry, 2003), is highly prevalent in adult animals (Weigler et al., 1990, 1993) but disseminated infection and severe disease are only rarely observed (Bailey and Miller, 2012; Carlson et al., 1997; Daniel et al., 1975; Dugan et al., 2013; McClure et al., 1973; Scharf et al., 2008; Simon et al., 1993) and may be linked to a compromised immune system (Bailey and Miller, 2012; Carlson et al., 1997; McClure et al., 1973; Scharf et al., 2008). In sum, simplex viruses of humans and NHPs are highly adapted to their natural hosts due to extensive co-evolution and rarely cause severe disease.

Although HSV-1 and HSV-2 infection of humans is mostly asymptomatic or associated with mild symptoms, these viruses can induce a lethal disease in certain NHPs, including marmosets (*Callithrix jacchus*) (Costa et al., 2011; Hatt et al., 2004; Imura et al., 2014; Longa et al., 2011; Mätz-Rensing et al., 2003). Conversely, transmission of BV from macaques to humans via bites, scratches or exposure to contaminated urine and feces can cause encephalitis (Elmore and Eberle, 2008; Hilliard, 2007; Huff and Barry, 2003). After the first description of BV infection and ensuing encephalitis in a laboratory worker in the 1930s (Gay and Holden, 1933; Sabin and Wright, 1934), about 40 human infections were reported. Before the availability of antiviral therapy, roughly 80 % of these infections had a fatal outcome (Elmore and Eberle, 2008; Hilliard, 2007; Huff and Barry, 2003). In contrast, no human infections with the HSV analogues of baboons, herpesvirus papio 2 (HVP-2, *Papiine herpesvirus 2*), and African green monkeys, Simian agent 8 (SA8, *Cercopithecine herpesvirus 2*), were reported and it is believed that these viruses might be apathogenic in humans. The reasons for the presumed differential pathogenicity of BV, HPV-2 and SA8 in humans are unclear.

In light of the severe threat to human health posed by BV, it is important to establish diagnostic tools that allow highly sensitive detection of antibodies against this virus. Moreover, diagnostic tests should be highly specific, since antibody responses against HSV and BV need to be discriminated in BV-exposed humans. Approaches to attain these goals face several challenges: Generation of large volumes of BV-infected culture supernatants for diagnostic purposes may pose a risk for accidental infection, and work with BV in the US and UK is restricted to BSL4 laboratories. Moreover, simplex viruses of humans and macaques share a high degree of sequence homology and antibodies are frequently cross-reactive (Elmore and Eberle, 2008). Finally, antibody responses against BV in infected macaques may be established late after infection and may vary significantly over time (Elmore and Eberle, 2008; Hilliard, 2007; Hilliard and Ward, 1999). Previous studies addressed safety concerns regarding work with BV by using HSV, HPV-2 and SA8 as antigens for sensitive detection of antibody responses against BV but a systematic comparison of these antigens has not been reported (Eberle and Hilliard, 1989; Fujima et al., 2008; Heberling and Kalter, 1986; Ohsawa et al., 1999; Takano et al., 2001; Yamamoto et al., 2005). Moreover, recombinant viral surface proteins were employed for detecting BV-directed antibodies and evidence was reported that this approach might allow BV-directed responses to be discriminated from HSV-directed responses (Fujima et al., 2008; Perelygina et al., 2005). However, confirmation of these results by independent studies is largely lacking.

Here, we compared the suitability of HSV-1-, HSV-2-, SA8- and HPV-2-infected cells to sensitively detect BV-specific humoral responses, and we tested whether purified viral glycoproteins, BV gD, HSV-1gG and HSV-2 gG, allow us to discern between antibody responses raised against BV and HSV-1/HSV-2.

## 2 Material and methods

### 2.1 Cell culture

Vero and 293T cells were grown in Dulbecco's Modified Eagle's Medium (PAA Laboratories), supplemented with 10 % fetal bovine serum (Biochrom) and antibiotics (penicillin/streptomycin, PAA Laboratories). For subculturing and seeding, 293T cells were detached by resuspension in fresh culture medium and Vero cells were detached by using trypsin/EDTA (PAA Laboratories), respectively. 9E10 hybridoma cells (Evan et al., 1985) were grown in RPMI1640 supplemented with 10 % fetal bovine serum (Biochrom) and antibiotics (penicillin/streptomycin, PAA Laboratories) and diluted 1 : 10 for subculturing.

### 2.2 Viruses

HSV-1, laboratory strain HSV-1 17syn+ (Brown et al., 1973), HSV-2, laboratory strain HSV-2 333 (Seth et al., 1974), HVP-2 and SA8 were amplified in Vero E6 cells and the development of a cytopathic effect was monitored by light field microscopy.

### 2.3 Plasmids

The expression of a truncated form of BV gD, which encompasses the N-terminal 332 amino acids of gD and lacks the transmembrane domain, results in gD secretion into culture supernatants (Tanabayashi et al., 2001). To generate such an expression plasmid, BV gD was PCR amplified with primers SacI-Kozak-S1 BV gD (5${}^{\prime }$-GAGCTCACCATGGGGCCCGGCATCGCCGCG-3${}^{\prime }$) and XhoI-His-Myc-XbaI-A 996 BV gD (5${}^{\prime }$-CTCGAGCTAA TGATGATGATGATGATGCAGATCCTCTTCTGAGATG AGTTTTTGTTCTCTAGAGGGGCCCTGGATGGTGACG TC-3${}^{\prime }$) and inserted into plasmid pCAGGS (Niwa et al., 1991). Oligonucleotide XhoI-His-Myc-XbaI-A 966 BV gD added the sequence for a C-terminal Myc-6xHis antigenic tag, which allowed for the convenient detection of protein expression. For the expression of the N-terminal 191 amino acids of HSV-1 gG, PCR was performed with primers SacI-Kozak-S1 HSV-1 gG (5${}^{\prime }$-GAGCTCACCATGTCGCAGGGCGCCATGCG) and XbaI- A 573 HSV-1 gG (5${}^{\prime }$-TCTAGAGGTGTCCAGGGCGGGG GAGGC-3${}^{\prime }$). For construction of secreted HSV-2 gG, the sequences encoding the N-terminal 23 amino acids (predicted signal peptide of HSV-2 gG) were fused to the sequences encoding amino acids L343 to D650 of HSV-2 gG employing primers SacI-Kozak-S-SP/1027 HSV-2 gG (5${}^{\prime }$-GAGCTCACCATGCACGCCATCGCTCCCAGGTTGCTT CTTCTTTTTGTTCTTTCTGGTCTTCCGGGGACACGC GGCGGGCTCATGGCCTTGACCGAGGAC-3${}^{\prime }$) and XbaI- A 1950 HSV-2 gG (5${}^{\prime }$-TCTAGAATCGAGAGCAGGGGA GGCCGTTAG-3${}^{\prime }$). The PCR-amplified HSV-1 gG and HSV-2 gG fragments were inserted into pCAGGS via SacI and XbaI. The integrity of all PCR-amplified sequences was verified by sequencing.

### 2.4 Serum samples

Human sera were selected from samples submitted to the Virology Department at the University Hospital Erlangen which had been tested for herpes simplex IgG in routine diagnostics by a commercial HSV-1/2 ELISA assay (ETI HSV-1/2 IgG, DIA-SORIN) and an immunoblot for type-specific antibodies (recomLine HSV-1 and HSV-2 IgG, Mikrogen). The serum panel included sera containing antibodies against HSV-1 and/or HSV-2 and sera negative for HSV antibodies. Serum samples of macaques were collected for routine screening for BV antibodies and were analysed either with an anti-HSV ELISA kit (Enzygnost^®^ Anti-HSV/IgG, Siemens) and/or the HVP-2 based ELISA described below.

### 2.5 Optimised Enzygnost^®^ Anti-HSV/IgG Kit

The screening of monkey sera for herpes B virus antibodies was initially performed by commercially available anti-HSV ELISA Kit (Enzygnost^®^ Anti-HSV/IgG, Siemens) as recommended by the manufacturer, with slight modifications. Modifications involved the replacement of the conjugate by rabbit anti-monkey IgG (H+L)-HRP (Nordic-MUbio) as described (Coulibaly et al., 2004).

### 2.6 Preparation of antigens and ELISA

Infection of cells and preparation of lysates was based on a previously described protocol (Ohsawa et al., 1999). In brief, Vero cells were seeded into 15 cm cell culture dishes and subconfluent monolayers were infected by HSV-1, HSV-2, HVP-2 or SA8 at low MOI. As a negative control, cells were mock infected. When cytopathic effects were observed throughout the monolayer, cell culture supernatant was removed and cells were washed with phosphate buffered saline (PBS). Subsequently, cells were covered with PBS/0.5 % Triton X-100 and detached using cell scrapers. Cell lysates were incubated on ice for 30 min and then clarified by centrifugation at 14 000 g for 30 s. The protein concentrations were determined by means of Pierce^™^ BCA Protein Assay Kit (ThermoFisher Scientific) according to the instructions of the provider. Cell lysates were adjusted to a concentration of 1 µg µL${}^{-1}$ by adding PBS/0.5 % Triton X-100 and stored at -80 ${}^{\circ }$C. For antibody screening, 12 × 8 U-bottom strip plates (Greiner bio-one) were coated with antigens diluted to 10 ng/well in a coating buffer (50 mM H${}_{2}$CO${}_{3}$ [pH 9.6], 20 mM Tris HCl [pH 8.5], 10 mM Na${}_{2}$HPO${}_{4}$ [pH 7.2], 1.4 mM KH${}_{2}$PO${}_{4}$ [pH 7.2], and 70 mM NaCl) and were incubated overnight at 4 ${}^{\circ }$C. Subsequently, the coating buffer was removed and unspecific binding sites were blocked by adding 3 % bovine serum albumin (BSA) dissolved in PBS for 1 h at 37 ${}^{\circ }$C. Wells were washed once with 1 x washing buffer (Candor Bioscience GmbH) and 0.2 mL diluent (1 % BSA and 0.1 % Tween 20 dissolved in PBS) was added. Thereafter, 20 µL of serum/diluent mixture were added per well and the wells were incubated for 1 h at 37 ${}^{\circ }$C. Subsequently, the wells were washed four times with 1× washing buffer. For detecting macaque antibodies, rabbit anti-monkey IgG (H+L)-HRP (Nordic-MUbio) and for detecting human antibodies goat anti-human IgG (H+L)-HRP (Dianova) were diluted 1 : 23 000 in diluent, added to the wells and the wells incubated 1 h at 37 ${}^{\circ }$C. Thereafter, the wells were washed four times with 1× washing buffer and incubated for 30 min at room temperature after adding 0.1 mL 1-Step^™^ Ultra TMB-ELISA Substrate Solution (ThermoFisher Scientific). The reaction was stopped by adding 0.1 mL 1 M H${}_{2}$SO${}_{4}$ and optical density was determined at 450 nm (Tecan Genios).

### 2.7 Glycoprotein expression and purification

293T cells were transfected by calcium-phosphate precipitation with pCAGGS plasmids encoding BV gD-Myc-His, HSV-1 gG-Myc-His, and HSV-2 gG-Myc-His, respectively. After overnight incubation in a humidified atmosphere at 37 ${}^{\circ }$C and 5 % CO2, cell culture medium was replaced by fresh FBS-free medium and incubated for an additional 24 h. Subsequently, cell culture medium was collected and stored at -20 ${}^{\circ }$C. The cells were maintained in fresh FBS-free medium and the collection of culture supernatants was repeated after an additional incubation period of 24 h. The recombinant glycoproteins were affinity purified from the culture supernatants by immobilised metal ion affinity chromatography (IMAC) on an ÄKTA avant FPLC system (GE Healthcare). The conditioned medium was cleared from dead cells and debris by sequential centrifugation for 10 min at 300 g and subsequently loaded to IMAC columns (HisTRAP Excel, 1 mL bed volume, GE Healthcare). Elution of bound proteins was performed by running a 10-column volume gradient with increasing imidazole concentration (0–300 mM) in PBS. 1 mL fractions were collected and monitored for protein contents and contaminations by recording UV absorption at 260 and 280 nm. Subsequently, glycoproteins were dialysed by means of Slide-A-Lyzer^®^ MIMI Dialysis Devices (10K MWCO; ThermoFisher Scientific) according to the instructions of the manufacturer. The concentration of dialysed proteins was determined by using Pierce^™^ BCA Protein Assay Kit (ThermoFisher Scientific). Proteins were dissolved (1 : 1) in glycerol and stored at -80 ${}^{\circ }$C. In parallel, aliquots were analysed by western blot and Coomassie staining. For western blot analysis, comparable amounts of the glycoproteins were separated by SDS-PAGE and transferred onto nitrocellulose membranes. Undiluted 9E10 hybridoma cell culture supernatant containing an anti-Myc antibody (Evan et al., 1985) was used as the primary antibody and a peroxidase-coupled anti-mouse antibody (1 : 10 000, Dianova) served as a secondary antibody. Signal detection was carried out in a ChemoCam imager together with the ChemoStar professional software (Intas) using 1 mL solution A (1.25 µM luminol sodium (Sigma) dissolved in 100 mM Tris [pH 8.6]), 100 µL solution B (6.7 µM p-comaric acid (Sigma) dissolved in DMSO), and 1.5 µL 3 % H${}_{2}$O${}_{2}$.

### 2.8 Immunoblot

For immunoblot detection of the presence of antibodies against alphaherpesviruses in human and macaque sera, identical amounts of BV gD, HSV-1 gG or HSV-2 gG were resuspended in 2× SDS-containing lysis buffer (50 mM Tris [pH 6.8], 10 % glycerol, 2 % SDS, 5 % β-mercaptoethanol, 0.1 % bromophenol blue, 1 mM EDTA) and boiled for 15 min at 96 ${}^{\circ }$C, separated by SDS-PAGE and transferred onto nitrocellulose membranes. Nitrocellulose membranes were blocked with 5 % milk powder in PBS-T (PBS with 0.1 % Triton X-100). After three washing steps with PBS-T, nitrocellulose membranes were cut to strips of approx. 4 mm in width. Afterwards, membrane strips were incubated for 1 h at room temperature with macaque or human sera (1 : 100 dilution in 5 % milk powder/PBS-T). After being washed three times for 10 min in PBS-T, membrane strips were incubated for 1 h at room temperature with secondary antibodies (1 : 2500 dilution in 5 % milk powder/PBS-T). Rabbit anti-monkey IgG (H+L)-Biotin (Nordic-MUbio) was used for detecting monkey antibodies and goat anti-human IgG (y-chain)-Biotin (Sigma) was used for detecting human antibodies. After three washing steps with PBS-T for 10 min, membrane strips were incubated for 1 h at room temperature with streptavidin-alkaline-phosphatase (Promega) that was diluted 1 : 5000 in PBS-T. Subsequently, membrane strips were washed three times for 10 min with PBS-T. A commercially available alkaline-phosphate substrate (BCIP/NBT (Plus); Moss, INC.) was used to detect bound antibodies. The reaction was stopped by rinsing membrane strips with H${}_{2}$O.

## 3 Results

### 3.1 SA8 and HPV-2 antigens allow for efficient detection of antibodies generated against BV

**Figure 1:**
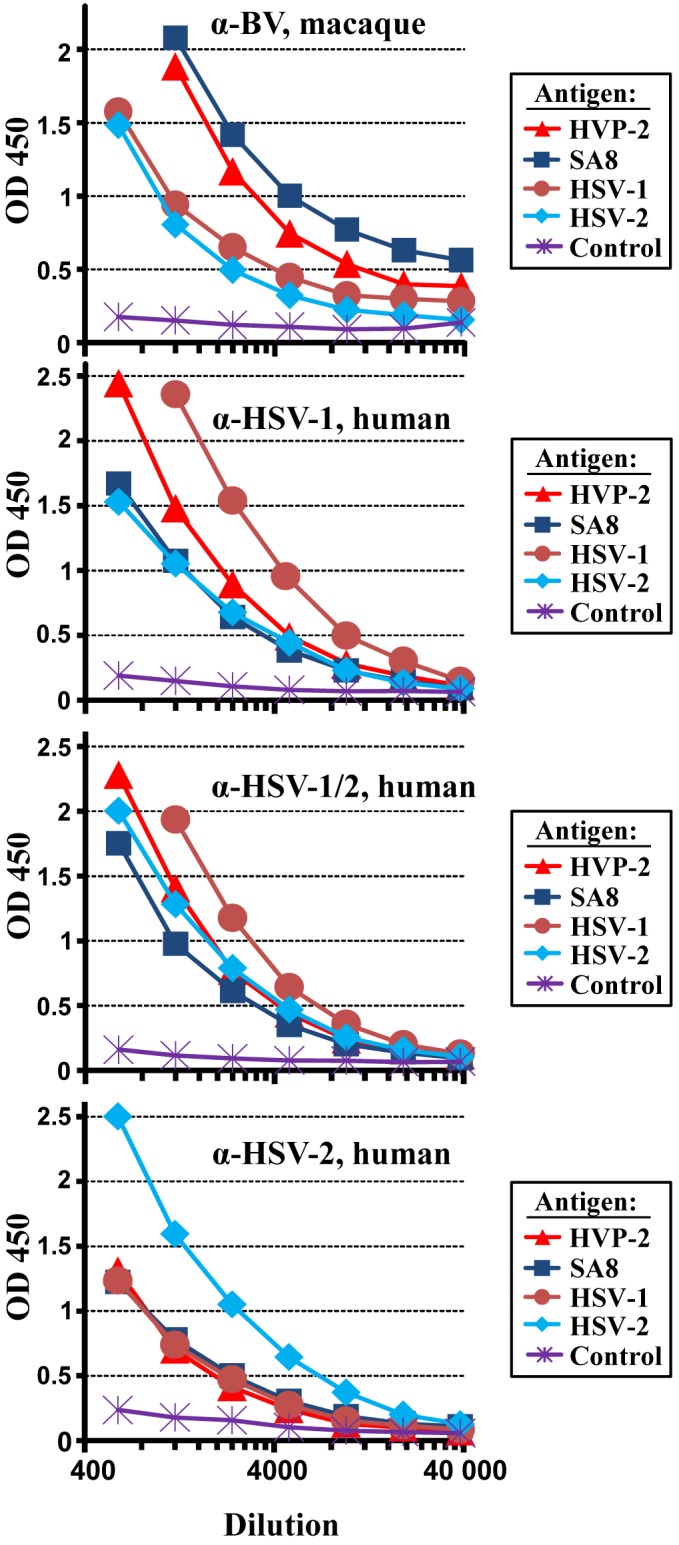
HVP-2 and SA-8 antigens allow for a more sensitive detection of antibodies raised against BV than HSV antigens. Reactivity of BV antibody-positive macaque serum M 2675 (α-BV, macaque), HSV-1 antibody-positive human serum H 2391 (α-HSV-1, human), HSV-1/2 antibody-positive human serum H 4337 (α-HSV-1/2, human) and HSV-2 antibody-positive human serum H 8935 (α-HSV-2, human) with lysates prepared from HVP-2, SA8, HSV-1, HSV-2, and uninfected Vero cells (control) was analysed by ELISA. Optical density (OD 450 nm, y axis, linear) measured for 2-fold serially diluted sera was plotted against the serum dilution factor (x axis, logarithmic).

To develop a sensitive in-house test for simplex-virus-specific antibodies in macaques, we first compared the cross-reactivity of previously characterised human and macaque sera with SA8-, HPV-2, HSV-1 or HSV-2 antigens. To this end, we infected Vero cells with these viruses at low MOI, and prepared cell lysates after the infections had caused cytopathic effects to a comparable extent. Thereafter, we coated plates with identical protein concentrations for subsequent ELISA-based antibody detection. In this test system, a macaque serum, known to react against BV bound robustly to lysates from HPV-2- and SA8-infected cells while binding to control lysates from uninfected cells, was inefficient (Fig. 1). The reactivity with lysates from HSV-1- and particularly HSV-2-infected cells was reduced compared to lysates from HPV-2- and SA8-infected cells. Increased reactivity with the cognate antigen was also observed for the other sera tested: sera from HSV-1- and HSV-2-infected patients reacted most efficiently with HSV-1 and HSV-2, respectively, and an intermediate phenotype was observed with a serum known to be reactive against both HSV-1 and HSV-2 (Fig. 1). These results indicate extensive cross-reactivity of antibodies raised against simplex viruses of humans and macaques. Moreover, our observations suggest that HPV-2 and SA8 antigens are suitable for detecting antibodies raised against BV, in keeping with published data (Ohsawa et al., 1999; Takano et al., 2001; Yamamoto et al., 2005). Since no marked difference was observed in the reactivity of macaque sera with SA8 and HPV-2, HPV-2 was arbitrarily chosen for subsequent analysis.

### 3.2 HVP2-based ELISA allows for detecting antibodies raised against BV with higher sensitivity than a HSV-1-based ELISA

**Figure 2:**
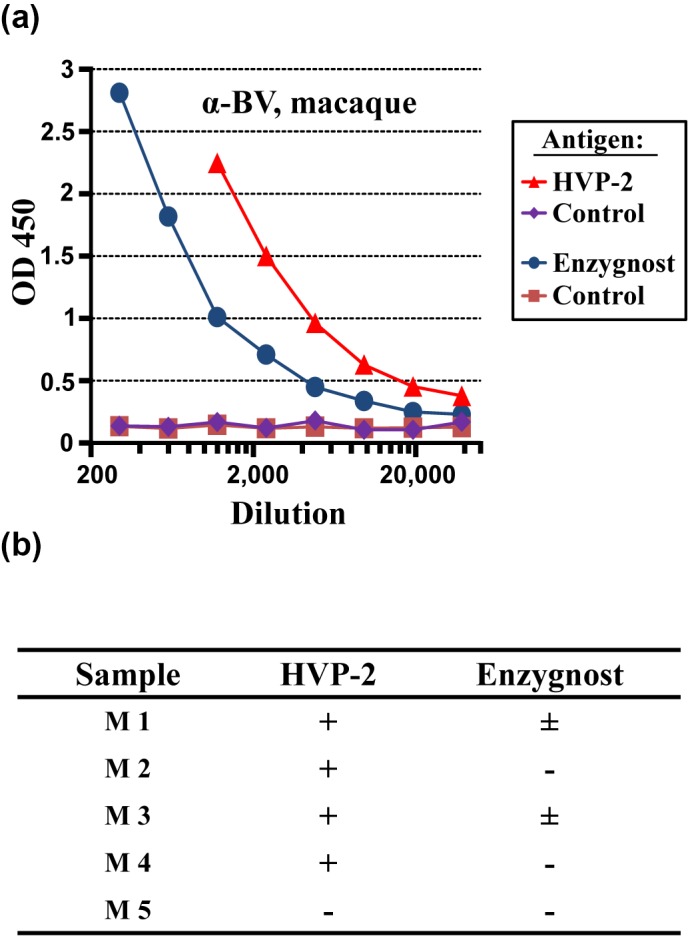
HPV-2-based ELISA allows for a more sensitive detection of antibody responses against BV than a commercial HSV ELISA. **(a)** Reactivity of BV-antibody-positive macaque serum M 2675 (HVP-2, Enzygnost) and BV-antibody-negative macaque serum M 16322 (control) against HPV-2 lysates and Enzygnost Kit-coated wells was examined. The optical density (OD 450 nm, y axis, linear) of 2-fold serially diluted sera are plotted against serum dilution factor (x axis, logarithmic). **(b)** Sera from cynomolgus macaques diluted 1 : 100, 1 : 150 and/or 1 : 300 were tested in the HPV-2-based ELISA or the optimised Enzygnost Kit. Results were assessed as follows: negative (-): ratio between reactivity with infected cells and uninfected cells below 2.5-fold, equivocal (±): ratio between 2.5 and 5-fold, and positive (+): ratio above 5.

We next compared sensitivity of the HPV-2-based ELISA with a commercially available system (Enzygnost^®^ Anti-HSV/IgG, Siemens) that was optimised for detecting BV antibodies in macaques as described previously (Coulibaly et al., 2004). A side-by-side comparison of both ELISAs with identical secondary antibody revealed a higher sensitivity of the HPV-2-based assay (Fig. 2a), in keeping with the results obtained with lysates of infected cells (Fig. 1). Moreover, several macaque sera diagnosed as negative or equivocal employing the HSV-1-based assay were tested positive using HPV-2 antigen (Fig. 2b). These results indicate that lysates from HVP-2-infected cells might allow for detecting antibodies raised against BV with higher sensitivity than the optimised anti-HSV ELISA Kit.

### 3.3 Evidence that recombinant BV gD and HSV-1/2 gG might allow for specific detection of antibodies raised against BV

**Figure 3:**
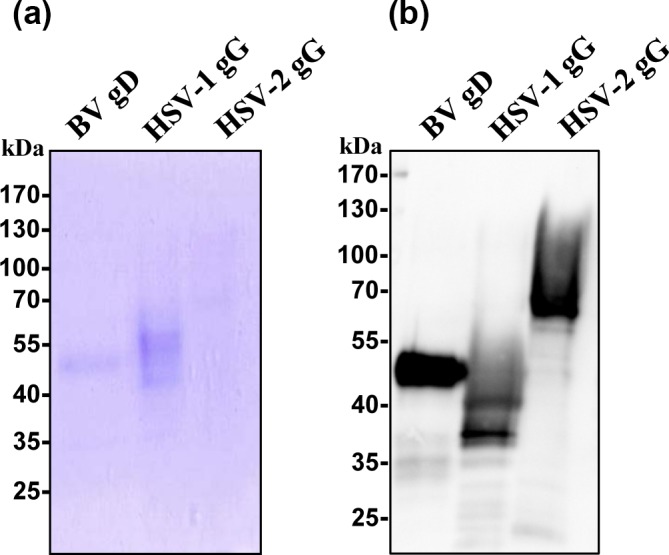
Analysis of purified viral glycoproteins. Equal amounts of recombinant gD of BV (BV gD), gG of HSV-1 (HSV-1 gG) gG of HSV-2 (HSV-2 gG) were separated on SDS-PAGE and visualised by Coomassie blue stain **(a)** or detected by anti-myc antibody after being separated by SDS-PAGE and transferred onto a nitrocellulose membrane **(b)**.

The surface proteins of herpesviruses mediate entry into host cells and are targets for neutralising antibodies. Variation in the glycoprotein sequences between simplex viruses of humans and NHPs might allow for detecting antibodies against these viruses with high specificity. We tested this concept employing BV gD and HSV-1 and HSV-2 gG, since a comparable approach has previously been documented (Fujima et al., 2008). To generate recombinant proteins, glycoprotein variants lacking the transmembrane domain but harbouring a C-terminal myc-His-antigenic tag were transiently expressed in 293T cells and purified from culture supernatants. Gel-electrophoresis and staining with Coomassie blue as well as western blot analysis revealed that proteins of the expected sizes were expressed and that purification was efficient (Fig. 3). It should be noted that the relatively low signal for HSV-2 gG upon staining with Coomassie blue was likely due to the presence of glycoforms with a heterogeneous molecular weight.

**Figure 4:**
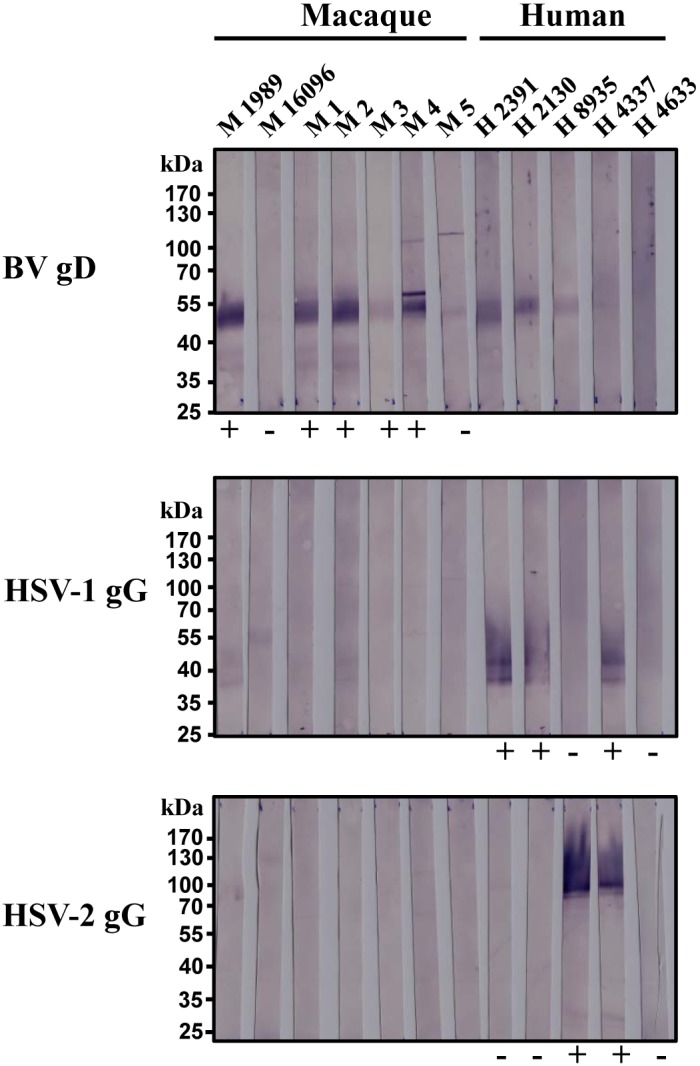
Evidence that recombinant BV gD and HSV-1/2 gG might allow for a discrimination of antibody responses against BV and HSV-1/2 in macaques. Reactivity of macaque and human sera with gD of BV (BV gD), gG of HSV-1 (HSV-1 gG), gG of HSV-2 (HSV-2 gG) immobilised on nitrocellulose membranes was examined. Macaque sera were selected for analysis based on reactivity with HPV-2 in the ELISA format. Additionally, human sera which had been tested for HSV-1/2 in routine diagnostics by a commercial ELISA assay (ETI HSV-1/2 IgG, DIA-SORIN) and an immunoblot for type-specific antibodies (recomLine HSV-1 and HSV-2 IgG, Mikrogen) were used. Results of previous tests with HPV-2 ELISA (macaque sera) or recomLine HSV-1 and HSV-2 IgG immunoblot (human sera) are depicted as negative (-) or positive (+) at the bottom of corresponding immunoblots. Designations at the head depict the source and nomenclature of sera.

Immunoblot analysis employing the recombinant proteins for detecting simplex virus-specific antibodies revealed a strong reaction of several macaque sera with BV gD. Moreover, two human sera from HSV-1-positive individuals and a serum from an HSV-2-positive human donor reacted with gD (Fig. 4). In contrast, none of the macaque sera reacted appreciably with gG of HSV-1 and HSV-2 origin while these proteins were efficiently recognised by sera obtained from HSV-1- and/or HSV-2-infected humans (Fig. 4). These observations suggest that antibodies induced upon HSV-1 infection may cross-react with BV gD while the humoral response to BV infection might rarely encompass antibodies that cross-react with HSV-1/2 gG. As a consequence, the combination of BV gD and HSV-1/2 gG might be suitable to determine whether macaques developed antibody responses against BV or HSV-1/2 but cannot be used to discriminate whether human patients developed antibodies against BV or HSV-1/2.

## 4 Discussion

The prevention of human exposure to BV, specific detection of BV infection of humans and establishment of BV-free macaque colonies (Yee et al., 2016) depend on the availability of diagnostic systems, which allow for the detection of antibodies against simplex viruses of human and NHP origin with high sensitivity and specificity. Our study shows that SA8- and HPV-2-infected cells allow for the detection of antibodies raised against BV with higher sensitivity than HSV-1- and HSV-2-infected cells. Moreover, we provide evidence that antibodies elicited against BV efficiently bind to BV gD but not HSV1/2 gG while antibodies produced in response to HSV-1 infection may cross-react with BV gD.

The high virulence of BV in humans calls for efforts to exploit less pathogenic simplex viruses of human and NHP origin for the detection of antibody responses to BV infection. This approach makes use of cross-reactivity of antibodies and was previously employed by several studies (Ohsawa et al., 1999; Takano et al., 2001; Yamamoto et al., 2005). They demonstrate that SA8- or HPV-2-infected cells allow for detecting humoral responses against BV with largely the same efficiency as authentic BV antigen. In contrast, lysates from HSV-1-infected cells were slightly less efficient (Ohsawa et al., 1999). The present study essentially confirms these findings and suggests that SA8 and HPV-2-based systems might allow to detected antibodies raised against BV with comparable efficiency. Collectively, use of BV-related simplex viruses for diagnostic purposes allows convenient detection of humoral responses against BV with high sensitivity and with modest cost and is thus suitable for colony screening and related purposes.

BV and human simplex viruses encode eight major glycoproteins (gB, gC, gD, gE, gG, gH, gI and gL), which are inserted into the viral envelope and facilitate attachment of virions to target cells and fusion of the viral membrane with a target cell membrane (Perelygina et al., 2003). Several of these glycoproteins encode regions which vary significantly between simplex viruses, and the use of recombinant proteins might thus allow discriminating humoral responses raised against BV and human simplex viruses. Two previous studies exploited this approach. Fujima and colleagues reported that sera from BV-positive macaques react with BV gD but not HSV-1/2 gG while the reverse observation was made for sera from HSV-1/2-positive individuals (Fujima et al., 2008). Perelygina and coworkers documented that gB, gC, gD and the membrane associated from of gG (mgG) of BV origin allow for efficient detection of antibody responses to BV infection (Perelygina et al., 2005). Moreover, analysis of human sera revealed that antibodies generated in response to HSV-1/2 infection usually do not cross-react with BV mgG and gC (Perelygina et al., 2005). Our results are in agreement with the observation by Fujima and colleagues that sera from BV-positive macaques do not cross-react with HSV-1/2 gG and confirm that BV gD is a useful antigen for the sensitive detection of antibody responses to BV infection. In fact, sensitivity of the BV gD-based system was higher than that of the HVP-2-based assay (please compare reactivity of serum M5 in Figs. 2b and 4). However, our data also suggest that BV gD can be recognised by human sera that are reactive with HSV-1 and thus might not be suitable to discriminate whether antibody responses were elicited against HSV-1 or BV.

In sum, our study shows that both SA8 and HPV-2 can be used as antigens for detecting humoral responses against BV and that recombinant BV gD and HSV-1/2 gG can be employed to discriminate whether a macaque was infected with BV or HSV. Further studies are required to identify combinations of recombinant proteins allowing to discern whether a HSV-1-positive human patient was subsequently infected by BV. Recombinant BV gC and mgG might be suitable for these endeavours (Perelygina et al., 2005).

## 5 Data availability

All relevant data are presented in the manuscript. For additional information, please contact the corresponding authors.
